# Air Pollution and Settlement Intention: Evidence from the China Migrants Dynamic Survey

**DOI:** 10.3390/ijerph19084924

**Published:** 2022-04-18

**Authors:** Xiao Yu, Jianing Liang, Yanzhe Zhang

**Affiliations:** 1 Northeast Asian Research Center, Jilin University, Changchun 130012, China; yux@jlu.edu.cn; 2 Northeast Asian Studies College, Jilin University, Changchun 130012, China; liangjn19@mails.jlu.edu.cn or

**Keywords:** air pollution, air quality index, migrants, socioeconomic status, educational level, income, settlement intention

## Abstract

This study analyses the effect of air pollution on the settlement intention of migrants in China. In recent years, the willingness of residents to migrate induced by air pollution has received a lot of attention from academics. By matching information from the China Migrants Dynamic Survey from 2015 to 2017 with the air quality index (AQI), we used the Probit model to assess the impact of air pollution on the settlement intentions of migrants with different socioeconomic statuses. First, we demonstrated that air pollution has a significant negative effect on migrants’ settlement intention. Second, we found that the effect of air pollution on settlement intention is influenced by individual socioeconomic status; that education level, as an indicator of cognitive ability, affects migrants’ motivation to migrate; and that personal income, as an indicator of economic ability, affects the feasibility of their migration. Motivation to migrate and the feasibility of moving determine together the divergence in settlement intention, and those with higher incomes and higher education levels are more likely to leave cities with serious air pollution. Third, the heterogeneous effects suggested that the negative effect of air pollution was greater for older, male, and married migrants. Our findings suggested that air pollution has a variety of effects on the heterogeneous migrants, resulting in changes in the demographic structure of cities.

## 1. Introduction

**Figure 1:**
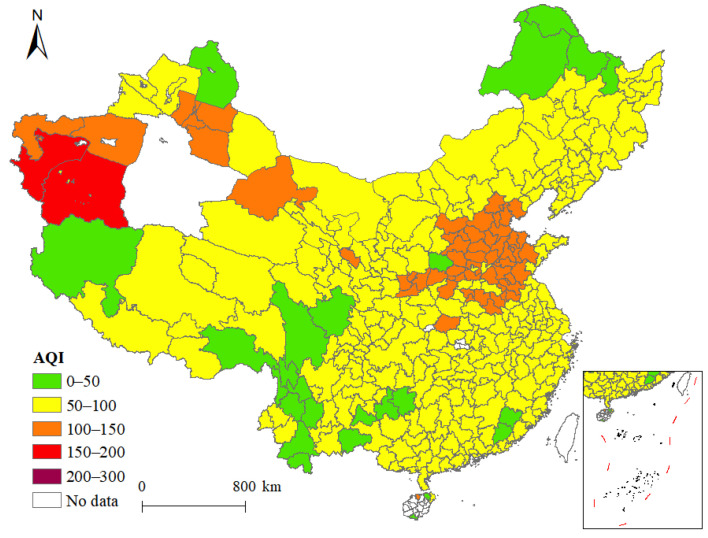
Spatial distribution of AQI of cities in 2017.

Since the Opening of China in 1978 and the rapid economic development that followed, the problem of environmental pollution has gradually emerged. Most parts of China have experienced it, albeit to varying degrees. The 2013 tract ‘Towards an Environmentally Sustainable Future Report’ stated that “among the 500 largest cities in China, fewer than 1% follow the World Health Organization air quality requirements, and seven out of the most 10 polluted cities in the world are in China” [1]. Although air quality has improved since the revision of the Air Pollution Prevention and Control Action Plan in 2013, most cities still fail to meet air quality standards. The distribution of the average annual Air Quality Index (AQI) in Chinese cities in 2017 is shown in Figure 1. From the figure, we found that air quality varied greatly between cities, such as Beijing, Tianjin, Hebei, Shandong, and Xinjiang, that central cities have poorer air quality, and that the eastern coastal region has better air quality overall.

Air pollution causes serious damage to both the physical and mental health of residents, and leads to a significant increase in respiratory diseases and lung cancer [2]. The inhalation of PM2.5 particulates (particles with a diameter of 2.5 µm or less) induces inflammation of the respiratory system and oxidative stress response [3], and the presence of PM10 particulates (particles with a diameter of 10 µm or less) in the air have been shown to have a significant correlation with respiratory mortality [4]. Air pollution also increases the mortality rate of people with heart and lung diseases, which significantly hinders the expected trend of improved health and extended life expectancy among the population [5]. The health effects of air pollution, however, are not only physical: it is common for affected residents to experience a rise in anxiety and other unwanted psychological afflictions [6]. Long-term exposure to air pollution can also increase the incidence of persistent depression [7]. In the short term, residents can minimize the damage caused by air pollution by avoiding or reducing outdoor activities or by wearing a mask. Residents with a longer view, however, are gradually choosing to migrate to cities with better air quality, and as a result, air pollution has become an important factor in determining their settlement intentions.

As an important component of urban environmental quality and livability, the effect of air pollution on the labor force has also received much attention in recent years, and studies have found that air pollution reduces the labor supply [8,9]. Air pollution also leads to a decrease in labor productivity [10]. Air pollution is one of the important causes of population migration changes in China and causes of labor outflow. However, most existing studies have been analyzed using aggregated data at the provincial or municipal level to examine the impact of air pollution on labor mobility at the macro level. In fact, labor mobility and supply at the macro level are the results of individual decisions at the micro level, so it is important to look not only at the impact of air pollution on regional labor mobility and labor supply at the macro level, but also at the mechanism of air pollution’s impact on individual labor migration decisions at the micro level. On the other hand, most of the existing studies are analyzed under the assumption of migrants homogeneity, ignoring the heterogeneity of migrants. The heterogeneity of migrants refers to the differences in individual characteristics of migrants, including gender, age, education level, and personal income. Differences in these characteristics directly affect not only their migration decisions and settlement intentions but also their value judgments about the macro factors that influence their decisions. In this paper, we focus on the heterogeneity of migrants in terms of education level and personal income. The causes of migration are the combined effects of push and pull, but due to differences in individual characteristics, migrants with different levels of education and different incomes perceive the push and pull differently and assess the push and pull factors with different criteria [11,12]. Therefore, there are differences in the effects of macro factors on the migration decisions and settlement intentions of migrants with different characteristics, i.e., the heterogeneity of migration effects. This paper is concerned with the different effects of air pollution on the settlement intentions of migrants with different levels of education and personal income. The different migration decisions made by migrants with different levels of education and income in the face of air pollution will ultimately affect the demographic structure of the city. If the exodus of people with high education levels and high incomes continues, air pollution will eventually cause a sinking demographic structure. Further in-depth research is needed to address these limitations in the literature. Therefore, our paper focuses on the extent to which air pollution affects migrants’ settlement intention. Does migrants’ settlement intention diverge due to differences in individual socioeconomic status? What are the theoretical and policy implications?

This paper focuses mainly on the impact of air pollution on the settlement intention of migrants, which has important practical significance. China has a unique *Hukou* system, which is the residential registration system. The *Hukou* system is key to understanding the migrant issue in China. There are differences between those with and without *Hukou* in terms of urban public services, housing segregation, and education for children [13,14]. In China, migrants without *Hukou* in their destination cities are called the floating population, and migrants who have been granted *Hukou* in their destination cities are called permanent migrants [15]. In our study, migrants refer to those without *Hukou* in their destination cities, also called the floating population. The number of migrants in China is huge and growing yearly, from 14,449 million in 2000 to 22,143 million in 2010. The number of migrants in China reached 376 million in 2020, accounting for 26.6 percent of the country’s total population according to the Seventh Census of 2020. The large-scale migrants provide the labor supply for the inflow cities and promote urban economic development. The population is the core resource of sustainable economic development in cities, and cities need to attract new migrants. Enticing migrants to stay is a key factor in the population competition between cities. A full understanding of factors that influences the settlement intention of migrants will help city governments to formulate policies that will attract migrants to settle, increase the local labor supply and promote local economic development. Second, as the originally-registered population of city age, new migrants are needed to balance the demographics. Attracting migrants to settle is of far-reaching significance to alleviate regional aging and improve the population structure. Last, in the COVID-19 era, the retention of migrants to alleviate the ensuing structural employment problems (which were amplified by the epidemic) has become a key determinant in achieving rapid economic recovery in cities. Therefore, our research focuses on how migrants who have entered a particular city can be persuaded to settle down.

Based on the above analysis, an individual utility function was constructed to analyze the impact of air pollution on migrants’ settlement intention. The Air Quality Index (AQI) released by the China National Environmental Monitoring Center was used to measure levels of air pollution. This was then combined with the dynamic monitoring data in the China Migrants Dynamic Survey (CMDS) from 2015 to 2017 to estimate the influence of air pollution on heterogeneous migrants through the IV Probit model. We found that first, air pollution has a significant negative effect on migrants’ settlement intention when other urban characteristics are controlled; second, the effect of air pollution on migrants’ settlement intention varies depending on individual education level and personal income (that migrants with high income and education levels are more likely to leave cities with severe air pollution); and third, the negative effect of air pollution is greater for older, male and married migrants.

This study can provide unique supplemental contributions in three aspects. First, it combines with the classical push-pull theory and individual utility function, fully considers the heterogeneity of migrant groups, and investigates the influencing mechanism of individual settlement intention from the micro level considering the heterogeneity of migrants. Second, it estimates migrants’ cognitive ability to assess the effects of air pollution based on their level of education; and it uses personal income to represent the economic feasibility of migration. Through these points, we explain the effects of air pollution on the heterogeneous migrants from a theoretical perspective and analyze the socioeconomic status differentiation and mechanism of migration intention caused by air pollution. Third, the study found that those with higher incomes and education levels were more likely to emigrate from cities with serious air pollution. This indicates that air pollution could lead to changes in urban population structures and the sinking of the social class. Our findings have important practical significance for urban development: to attract skilled migrants. Policymakers must address the issue of air quality to create a comfortable and livable environment. Compared with the existing literature, this study highlights the divergent role of status differences measured by education level and personal income on the effect of air pollution on migrants’ settlement intention as a way to explore the effect of air pollution on the urban population, which enriches the existing studies to a certain extent and explains the differential effect of air pollution on heterogeneous migrants at a theoretical level.

## 2. Literature Review and Research Hypotheses

Population migration has been extensively and thoroughly studied in the literature and has been explored from several theoretical perspectives, such as human capital theory [16], assimilation theory [17], and push-pull theory [18]. The most influential one is the push-pull theory, which argues that migration is a combination of the push force of the emigrating city and the pull force of the immigrating city, and the population will make migration decisions by comparing the advantages of the immigrating city with the disadvantages of the emigrating city. The push-pull theory has good explanatory power for population migration, but migration into a city does not mean that settlement in that city can be completed. Unlike other countries, migration and settlement do not occur simultaneously in China, and population migration is neither one-step process, nor do they differ in important ways from the circular flows of other countries. Settlement intention is a unique research topic in the Chinese context, and Chinese scholars have conducted in-depth studies on the factors that influence migrants’ settlement intention. From an economic perspective, migrants tend to settle in a destination that meets their economic expectations [19]. Their settlement intention is significantly positively correlated with the ratio of income to expenditure, while high housing costs have a restraining effect [20]. In addition to economic factors, public services, and city amenities significantly enhance settlement intention [21,22,23]. From the perspective of individual characteristics of migrants, gender, marital status, age, and education level have a significant impact [24,25] Social integration, relative deprivation, and other psychological factors influence where migrants choose to live [26]. Social factors such as the length of time a migrant has lived in a city, the strength of their social relationships and networks also affect the intention to settle [27,28,29].

In recent years, the impact of air pollution on migration decisions and settlement intentions has gradually begun to emerge as air pollution intensifies and people aspire to a better life. Settlement intention can be understood as a re-decision to either settle down in the current city or continue to migrate. It is a measure of how satisfied migrants are with the result of their last migration after living in the destination for a period. Research into the impact of air pollution on settlement intention is based on the study of air pollution on the migration decision. Air pollution has a negative effect on labor, whereby workers will emigrate from a polluted area if they have the means and opportunity to do so. Air pollution does have a negative effect on the labor supply, with a 1% increase in sulfur dioxide emissions leading to a 0.028% decrease in labor supply [30]. Other scholars have confirmed this, with studies that prove better air quality attracts more new residents [31]. Another study found that air pollution causes significant changes to population inflow and outflow at the county level. Increased air pollution will reduce the country’s inflow by 50% and the population by 5% by net migration [32]. A 10% increase in the average PM2.5 index increases labor outflow by about 1% [33]. A study of Baidu’s migration index found that for every 100-point increase in the Air Quality Index (AQI), the number of people in that area who searched for “migration” on Baidu would increase by 2.3–4.7 percent [34]. Another study suggests that air pollution will reduce the stock of innovative technological professionals(TIP), and a 1% increase in PM2.5 in China’s cities decreases the stock of TIP by 146 people [35]. There are also views that in China, population loss caused by environmental pollution is currently only seen in economically developed coastal areas and large inland cities [36]. However, because China’s migrants come mostly from rural, low-income areas, it often ranks expected revenue higher than concerns about air pollution when deciding on a destination. This may mean that air pollution indirectly leads to more rural migrants moving to cities [37].

A review of the existing literature reveals that most existing studies have analyzed macro-level labor mobility caused by air pollution using macro-level data. However, the outcome of labor mobility at the macro level is determined by the decisions of numerous micro individuals, so it is necessary to analyze this at the micro level. On the other hand, migration decisions and settlement intentions are separated in China. Whether air pollution has a crowding-out effect on migrants’ settlement decisions has not been analyzed in depth. This study examines whether migrants who have already entered decide to settle there, and explores the impact of air pollution on the settlement intention of migrants at the individual level. As with labor migration decisions, migrants’ settlement intentions depend on the level of utility they have obtained in their current city compared with what they think they could obtain in other cities. Therefore, we constructed the personal utility function as:

(1) $$U_{ijt}=W_{ijt}-V_{ijt}{}_{\;}\;\;$$

where *U_ijt_* is the utility level of migrant *i* living in city *j* at time *t*, *W_ijt_* is the positive utility brought by wages, public services, etc., and *V_ijt_* is the negative utility brought by air pollution. Migrants choose to stay in their current city or move to other cities depending on the obtainable level of utility. If the utility level is higher in another city, migrants will leave the city where they currently reside: i.e., when *U_ijt_ < U_ikt_*, they migrate from *j* to *k*. Damage to health caused by air pollution brings dis-utility to migrants. The worse the air pollution, the greater the health effects, and the higher the dis-utility. Hypothesis 1 is therefore proposed:

**Hypothesis** **1** **(H1).** *Air pollution has a negative effect on migrants’ settlement intentions.*

From the information outlined in this section, we can see that research into the impact of air pollution on labor migration is based mainly on the premise of labor homogeneity analysis, which has largely ignored differences in the characteristics of individuals. In fact, migrants with different individual characteristics may make different decisions when faced with the same situation due to differences in their individual characteristics. Massey proposed that to fully explain migration behavior, it is necessary to consider the combined effects of the macro social structure, the micro individual, and the family decision-making process [38]. Takatoshi and Thisse introduced heterogeneity into the new economic geography model. They found that the labor did not respond uniformly to the wage gap because it exhibited varied mobility behaviors and could be affected by individual characteristics, family and marital status, and personal preferences [39]. Additionally, migrants with a higher level of education usually have an elevated human capital, are less affected by the labor market, have more diverse employment options, can more easily get better jobs and high incomes in numerous different cities, and can better integrate into life at their destinations [32]. It has been proved that more highly skilled migrants with more advanced social levels tend to have higher requirements for livability [40]. As a result, the subjective feelings of migrants regarding air pollution vary significantly according to their economic and social status [41].

The cognitive ability of migrants to perceive the hazards of air pollution depends largely on their level of education, which is related to their motivation to migrate because of air pollution. The more advanced the individual’s education, the more environmental knowledge they have, and the more considered their subjective opinion of air pollution [11,42]. Studies on air pollution and labor productivity show that the impact of air pollution on highly educated workers is more obvious than that of those who are less educated [43]. The dis-utility of air pollution on highly educated migrants is, therefore, greater, and these migrants have an increased motivation to leave cities with serious air pollution. The deterioration of air quality significantly reduces the life satisfaction of individuals with high education levels. Hypothesis 2 is therefore proposed:

**Hypothesis** **2** **(H2).** *The negative impact of air pollution on the settlement intentions of migrants is greater for those with a higher level of education.*

High personal incomes ease the budgetary constraints of potential migrants and increase migration feasibility. Those with high incomes have a strong economic capacity and face fewer financial constraints when deciding whether to migrate. Because they are better able to bear migration costs, their risk is lower. This shows us that when both high-income and low-income migrants are exposed to air pollution, the former can “buy fresh air”, and migrate to cities with better air quality, while the latter is economically disadvantaged and unable to migrate [44]. We can also see that low-income migrants are more concerned with employment opportunities than the quality of their environment, i.e., the income utility is greater than the utility of clean air. The higher a migrant’s income, the more seriously they will consider the effects of air pollution, and the more likely it will form part of their migration decision. The deterioration of air quality significantly reduced the life satisfaction of individuals in the high-income group. It was proved that they were usually willing to pay more to live somewhere with better air quality [45]. Hypothesis 3 is therefore proposed:

**Hypothesis** **3** **(H3).** *The negative impact of air pollution on the settlement intentions of high-income migrants is greater.*

## 3. Data and Methods

### 3.1. Data

The data used in this paper were taken from the China Migrants Dynamic Survey (CMDS) organized by the National Health Commission of China (NHC). The NHC has implemented a continuous cross-sectional survey by questionnaire every year since 2009, covering areas with a high concentration of migrants. The annual data of the whole mobile population in 31 provinces (autonomous regions and municipalities) were used as the basic sampling frame, and a stratified, multi-stage, Probability Proportionate to Size (PPS) method was adopted for sampling. Participants were migrants over 15 years of age who had been living in the destination cities for more than a month. The sample of the survey are migrants without *Hukou*. With an annual survey sample size of nearly 200,000 households. It is a large authoritative data for studying the issues of Chinese migrants and has the characteristics of strong representativeness and a large sample size. In our study, we combined the survey data from 2015, 2016, and 2017.

We calculated the average annual air pollution in each city using the China National Environmental Monitoring Center’s daily air quality index (AQI). The AQI is the most direct indicator of air quality besides subjective perception. To control factors that may affect migrants’ decisions, we drew upon city information from the Statistical Yearbook of Provinces and China City Statistical Yearbook.

The dependent variable in this paper is the settlement intention of migrants. We used the questions “Do you intend to live in the area long-term (5 years)?” from the CMDS 2015 and 2016 surveys” and “Do you intend to stay in the local area for some time to come?” and “If you intend to stay in the local area, how long do you expect to stay?” from the CMDS 2017 survey, to build our index. Respondents who answered “I do not plan to stay” in the 2015 and 2016 surveys were assigned a value of 0, while those who answered “I plan to stay” were 1. In the CMDS 2017 survey, respondents who answered “No” to the first question were assigned a value of 0. Those who answered “Yes” were assigned a value of 1 if they answered “6–10 years”, “more than 10 years”, or “indefinitely” to the second question, and 0 if they answered “1–2 years” or “3–5 years”. Those who answered “not sure” were excluded.

The core independent variable in this paper is the AQI, a non-linear dimensionless index representing the state of air quality. The higher the value, the more severe air pollution and the greater the detriment to physical and mental health. According to China’s Air Quality Certification Standards, which lists AQIs ranging from 0–50 as excellent, 51–100 as good, 101–150 as lightly polluted, 151–200 as moderately polluted, 201–300 as heavily polluted, and above 300 as seriously polluted.

In addition to independent and dependent variables, this study also controlled the variables of economic development, total population, medical resources, education resources, industrial structure, and the housing price to income ratio. Apart from these city characteristics, the control variables included migrants’ genders, ages, *Hukou*, marital status, education, personal incomes, migration time, migration ranges, and reasons for migration.

A description of the main variables and descriptive statistics are shown in Table 1. After matching with city-level data, 243,253 samples were retained. The mean value of settlement intention is 0.790, indicating that 79 percent of migrants plan to live in their current location for more than 5 years. The mean value of the AQI is 83.965, and the standard deviation is 21.802, indicating significant differences in air quality among cities.

### 3.2. Empirical Strategy

To verify the hypotheses, the Probit model was used to assess the effect of air pollution on migrants’ settlement intentions. We set our baseline econometric specification as:

(2) $$P(y_{ij,t}=1|AQI_{j,t},X_{i}{}_{,t}^{},Z_{j}{}_{,t}^{})=F(AQI_{j,t},\beta_{1})=\Phi (\beta_{0}+\beta_{1}AQI_{j,t}+\beta_{2}X_{i}{}_{,t}^{}+\beta_{3}Z_{j}{}_{,t}^{})$$

where a migrant’s settlement intention *settle_ij,t_* is a binary choice variable, defined as either 1 or 0. If the migrant stays in the current city of residence in year *t*, the value is 1; if he/she leaves the current city, the value is 0. *P(settle_ij,t_* = 1) is the probability that the migrant will settle down in the current city in year *t*. *AQI_j__,t_* is the air quality index of city *j* in year *t*. *X_i_* is a vector of the migrant’s control variables, which are age, gender, reason for migration, migration time, migration range, *Hukou*, marital status, education, and personal income. *Z_j__,t_* is the city’s characteristics, which are economic development, industrial structure, total population, medical resources, educational resources, and the housing price to income ratio. The impact of air pollution on a migrant’s settlement intentions is measured by the coefficient *β*_1._ If *β*_1_ is significantly negative, it means that the worse the air pollution in a city, the greater the probability that migrants will leave.

We used the Probit model to estimate migrants’ settlement intentions. Although we controlled for many urban and individual factors, there was still concern about endogenous due to complex unobservable variables and measurement errors. At the same time, however, the relationship between economic development and air pollution is endogenous, and a causal bidirectional relationship exists between migrants’ settlement intentions and air pollution. To solve this problem, previous studies have used thermal inversion frequency or ventilation coefficients as instrumental variables for air pollution to better identify causal effects [32,34,46]. However, it has also been shown in the literature that urbanization may also contribute to thermal inversions and that the heat island effect of urban economic development may itself lead to thermal inversions [47]. This directly threatens the exogenous hypothesis. Therefore, we use the ventilation coefficients as an instrumental variable. The ventilation coefficients can be used as an instrumental variable for air pollution. Because, on the one hand, a larger ventilation coefficient indicates greater air mobility, which throws air pollution farther away and thus reduces air pollution, satisfying the correlation assumption of an effective instrumental variable [48]. On the other hand, the ventilation coefficients are influenced by both wind speed and atmospheric boundary layer height, which are determined by complex meteorological systems and geographical conditions, thus satisfying the exogenous assumption of the effective instrumental variable [49]. The ventilation coefficients are constructed as follows:

(3) $$VC_{it}=WS_{it}\times BLH_{it}{}_{\;\;}$$

where *VC_it_* denotes ventilation coefficients, *WS_it_* denotes wind speed, and *BLH_it_* denotes boundary layer height. The wind speed *WS_it_* and *BLH_it_* were obtained from the latitude and longitude raster data published by the European Centre for Medium-Range Weather Forecasts(ECMWF). We further parsed the raster data in ArcGIS software (Environmental Systems Research Institute, Redlands, CA, USA) to obtain the Chinese city data.

## 4. Results

### 4.1. Baseline Results

Table 2 reports the effects of air pollution on the settlement intentions of migrants. The first column contains the results of the Ols regression and illustrates that the coefficient between air pollution and the settlement intention is 0.0002, which is significant at a level of 1%. The second column displays the Probit results and indicates that the higher the AQI, the greater the probability that migrants will stay in their current city of residence. The Ols and Probit findings are not consistent with Hypothesis 1 because air pollution is an endogenous variable. Cities with more serious air pollution tend to have developed economies with a high proportion of labor-intensive industries and a substantial demand for migrants.

**Figure A1:**
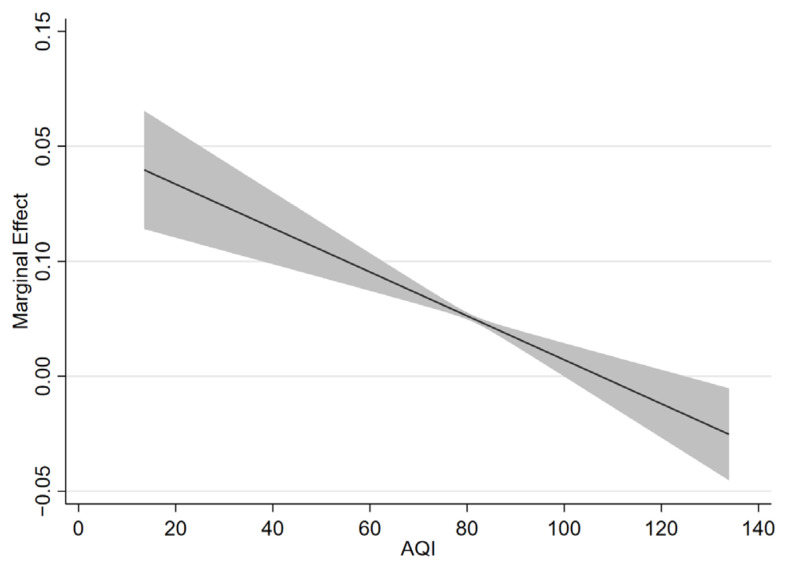
The marginal effect of AQI.

In this analysis, Ols and Probit, therefore, underestimate the effect of air pollution, and the reasons a migrant chooses to stay in a highly polluted city remain unclear. To address this problem, we used the ventilation coefficients as the instrumental variable of air quality. Column 3 of Table 2 shows the estimated results of the IV Probit model. The first stage regression shows a significant negative correlation between the ventilation coefficients and air pollution. The F-value of the first-stage regression is greater than 10, and there is no weak instrumental variable. The coefficient between air pollution and the settlement intention of migrants is −0.0632, which is significant at a level of 1%. The marginal effects of AQI (Figure A1 in the Appendix A) clearly show that when there is a negative effect of air pollution on migration, the marginal effect increases gradually as the AQI increases. This result indicates a significant negative correlation between air pollution and migrants’ settlement intention, and air pollution can significantly reduce the willingness of migrants to settle in their current living city. These findings support Hypothesis 1. With the addition of instrumental variables, the measurement error was stripped away, and the air pollution was reflected more truly. The error of latent variables was also corrected. Considering that the settlement intention of migrants in the current period does not affect historical air pollution, to mitigate the reverse causality bias, the AQI was regressed with a one-period lag, and the regression results show that the negative effects of air pollution on settlement intention still exist, and all are statistically significant, further verifying the causal relationship between air pollution and settlement intention.

The findings of the control variables suggest that the coefficient between GDP and migrants’ settlement intention is 0.2554, which is significant at a level of 1%. This result demonstrates that economic development has a significant attraction effect on migrants. Medical resources and educational resources represent the public services of the city, and their coefficients with the settlement intention of migrants are 0.0067 and 0.1566, respectively. Both are significantly positive at the 1% level, confirming that city public services attract migrants to settle long term. The coefficient between the tertiary sector’s share of GDP and migrants’ settlement intention is 0.0073. This is because tertiary industry provides many employment opportunities, which are an incentive for migrants to settle. The coefficients of population size and the ratio of housing price to settlement intention are both negative. The size of a city’s population reduces migrants’ intention to live in that city, as an overly dense population causes traffic congestion and difficulties in obtaining public services. With the increase in the housing price to income ratio, the more pressure on migrants to buy houses, the less their settlement intention.

### 4.2. Education Level and Personal Income

**Figure A2:**
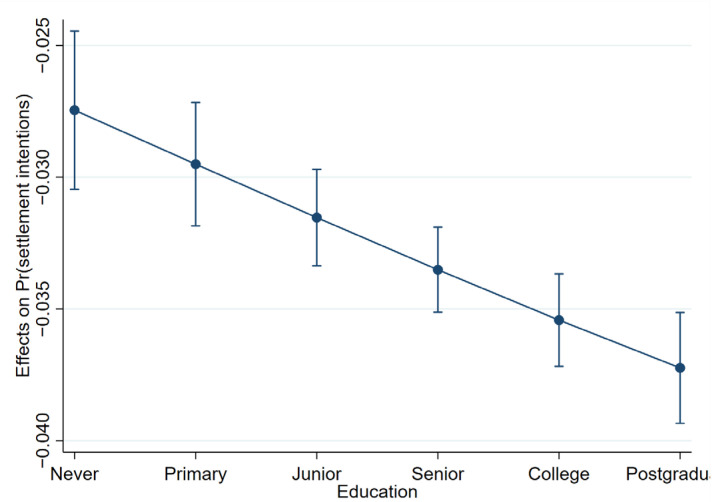
The average marginal effect of AQI at different levels of education.

To gain insight into the disparate effects of air pollution on migration, we further investigated whether pollution-related migrants’ settlement intentions differed among groups. We use the interaction term between AQI and education level to identify the variability in exposure to air pollution across education level groups. The regression results are shown in Table 3. The results show that the interaction coefficient between education level and air pollution is significantly negative at the 1% level, and education level has a significant moderating effect on the negative effect of air pollution. The higher the education level of migrants, the more likely they are to migrate because of air pollution. According to the regression results, we draw the marginal effect graph for intuitive analysis, as shown in Figure A2 in the Appendix A. The results clearly reflect that education level enhances the negative effect of air pollution on migrants’ settlement intention. The negative effects are not significant when migrants have a low level of education, but significant when migrants have a high level of education. Hypothesis 2 is verified. This may be because more high-education level migrants have an increased cognitive capacity to consider the damage to physical and mental health that it causes. Air pollution, therefore, has higher dis-utility for more highly educated migrants. Additionally, because they have higher human capital, high-educated migrants are less restricted by the labor market and can find satisfactory jobs in other cities. This means that high-education level migrants have more options and are more likely to migrate to avoid the damage from air pollution.

**Figure A3:**
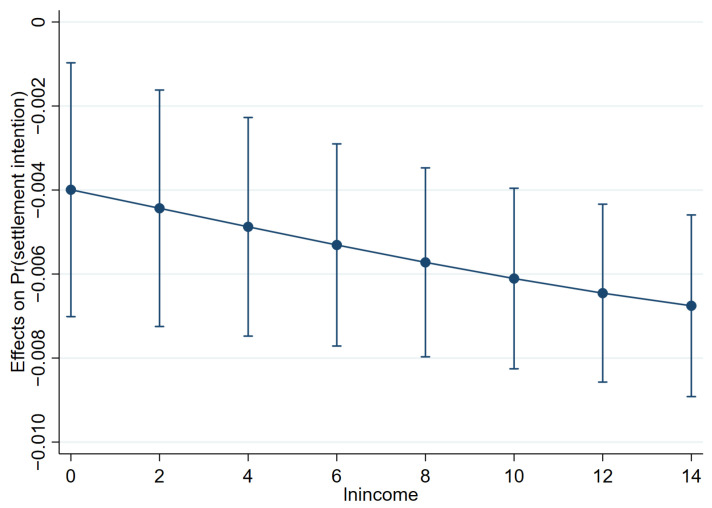
The average marginal effect of AQI at different income.

We then tested further whether the effect of air pollution on migrants’ settlement intention varies with personal income levels. We use the interaction term between AQI and personal income to identify the variability in exposure to air pollution for different upper income groups. In column 2, the interaction coefficient between migrants’ personal income and air quality is significantly negative at the 1% level, indicating that personal income regulates the effect of air pollution on migrants’ settlement intention. The higher the personal income of migrants, the greater the probability of migration due to air pollution is. According to the regression results, we draw the marginal effect graph for intuitive analysis, as shown in Figure A3 in the Appendix A. The results clearly reflect that personal income enhances the negative effect of air pollution on migrants’ settlement intention. The negative effects are not significant when migrants have a low level of personal income but significant when migrants have a high level of personal income. Hypothesis 3 has been verified. A possible reason for this is that the high-income migrants have fewer budgetary constraints and are more able to migrate. In many cases, they also possess accumulated capital, which allows them to cover migrating costs and mitigates the risk. Second, those with higher incomes can meet their basic needs relatively easily and think more about their quality of life. Those with higher personal incomes are therefore much more likely to migrate away from a city that has serious air pollution.

Our findings indicate that air pollution has a strong selective effect on the migrants’ settlement intention. Migrants with high incomes and high education levels are less likely to settle down in cities with serious air pollution. In contrast, because those with lower incomes and levels of education lack the ability to migrate, they are forced to stay in heavily polluted cities. In the long term, the outflow of high-income and high-education migrants in areas with elevated levels of air pollution will lead to a trend of localized backward social mobility. This trend has not yet manifested on a large scale, but as the residents demand better living conditions, it will become increasingly obvious. Because of its detrimental effect on cities and economic development, this trend may even lead to the phenomenon of counter-urbanization.

### 4.3. Robustness Test

Because city average annual AQI may mask seasonal variations and provide biased estimates, we implemented a robustness check to examine our results. We calculated the percentage of days with an AQI above 100, called the number of polluted days, as the independent variable with which to re-estimate. This classification is based on China’s Air Quality Certification Standards, which lists AQIs above 100 as polluted. The regression results are shown in Table 4. The results show that the more polluted days, the lower the settlement intentions of migrants, and the impact on migrants with higher education levels and higher personal income is greater. These regression results are consistent with the baseline results, which verify the robustness of the regression results.

### 4.4. Heterogeneity Analysis

To further examine the heterogeneity of the negative effect of air pollution, we first focus on the air quality differences in settlement intentions of different ages. We use the interaction term of AQI with age to identify differences between ages. The regression results are shown in Table 5. The results show that the coefficient of the interaction term is significantly negative. This indicates that the negative effect of air pollution becomes greater with increasing age. The negative effect of air pollution on young people is relatively low and is greatest for older people. There are several possible reasons for this. Young people might prioritize employment opportunities and development prospects and seek high wages. They also tend to be healthier, which may make them better able to resist the damage caused by air pollution. As individuals grow older, they gradually accumulate capital and focus more on quality of life. Additionally, people’s physical functions decline with age, making them more susceptible to the negative effects of air pollution. Older people are therefore extra sensitive to air pollution, both physically and psychologically, and are more likely to emigrate from polluted cities.

We use the interaction term of AQI and gender to identify the differences between genders. The results show that the coefficient of the interaction term is significantly negative (1 for males and 0 for females). This indicates that the negative effects of air pollution are greater for men. These results indicate that men are more likely to leave due to air pollution. A potential explanation is that men tend to spend more time engaging in outdoor activities (such as physical exercise), while women spend more time indoors, taking care of children and other family members. Consequently, men may be more likely to notice changes in air quality. These differences in exposure between men and women may explain their disparate attitudes toward air pollution as a factor in deciding where to live. Another reason is that the settlement decisions of Chinese families are usually made by men, so it is not surprising that the results show them to be more concerned about air pollution.

We use the interaction term between AQI and marital status to identify differences between marital status, and the results show that the coefficient of the interaction term is significantly negative (1 for married and 0 for unmarried). This indicates that the negative effect of air pollution is greater for the married group. The results indicate that those in the married group are more likely to migrate to avoid air pollution than those in the unmarried group. One possible interpretation is that the former considers the physical and mental damage that air pollution causes not only to their own health but also to that of their children and other family members, and this makes them more likely to settle in cities with better air quality.

## 5. Discussion

In recent years, air pollution has become the focus of the community’s attention. With the continuous improvement of residents’ demand for quality of life, people are more and more deeply aware of the harmfulness of air pollution and take various ways to avoid the negative impact of air pollution on personal health. Air pollution, as an important component of environmental quality and habitability, has an increasing impact on migration decisions. Under the background of China’s huge numbers of migrants, the floating population has brought abundant labor capital to the cities where it moves in. Increasing the willingness of migrants to settle is the key to urban development.

We examined this impact from a micro perspective using data from the China Migrants Dynamic Survey and distinguished the different negative effects of air pollution on heterogeneous migrants. Our results show that air pollution has a significant negative effect on migrants. We used migrants’ education level to represent their cognitive awareness of air pollution and their motivation to emigrate. Their personal incomes represented the economic feasibility of migration. The study proved that the negative effect is higher among those who are more educated and have higher incomes. The heterogeneous effects suggest that the negative effect of air pollution increases gradually with age, and men and married people are more likely to emigrate from polluted areas than women and unmarried people.

Previous studies on air pollution and migrants have focused largely on the impact of air pollution on migration decisions. Still, more important than the migration decision is whether migrants who have already moved to a city decide to settle there. Settlement intention can be understood as a re-decision to either settle down in the current city or continue to migrate. It is a measure of how satisfied migrants result from the last migration after living in the destination for a period. Settlement intention can more accurately reflect the evaluation results of the air quality of the city by migrants than migration decision. Our study demonstrates a significant negative effect of air pollution on settlement intentions. Combined with the existing literature, we can conclude that air pollution not only discourages migrants from entering the city but also has a crowding-out effect on migrants living in the city.

On the other hand, unlike previous studies that regarded migrants as homogeneous, our study focused on the differences among migrants and separately analyzed the heterogeneous effects of air pollution on migrants with different education levels and incomes. Our research suggests that highly educated and high-income migrants are more likely to migrate due to air pollution, in line with Maslow’s hierarchy of needs: once people have met their most basic physiological needs, they will begin to consider higher-level needs such as health and safety. Highly educated and high-income migrants will consequently seek better environmental and living conditions and as a result are more inclined to migrate to cities with better air quality. The study also found that air pollution is selective: less educated migrants with lower incomes do not have the means or opportunity to emigrate, so they will remain stuck in highly polluted areas.

Air pollution has a highly selective effect on migrants’ settlement intention, and the emigration caused by air pollution may occur more among people of middle and upper social classes. Those with the ability to migrate are usually those with higher education levels, more social resources, and abundant financial resources, while those facing difficulties migrating or having the inability to migrate are often relatively poor people without sufficient social network support. This is not only because the groups with higher education and income have a stronger perception of haze but also because these groups have a stronger ability to migrate and adapt. At the same time, air pollution also discourages some upper-middle-class people who might otherwise move in. Therefore, the difference in residence intention caused by air pollution will change the social class structure of the urban population. The subsidence of the urban population structure is not conducive to urban economic development and talent gathering.

Our findings also suggest that air pollution contributes to health inequalities, with the lower income and education groups at higher health risks due to their greater exposure to air pollution. Air pollution will be the important transmission mechanism of health inequalities because of the different income and levels of education concerning individual ability to evade environmental risk. The high-income and high-education groups have the ability to migrate to a city with better air quality and avoid air pollution damage. The low income and low education group lack the ability to migrate to other cities to avoid air pollution. Therefore, the different exposure levels of environmental pollution will become a new source of health and social inequality. This also means that improving urban air pollution is one of the important breakthroughs to reducing pollution exposure risk and achieving environmental health equity.

## 6. Conclusions

This paper focuses on the differences in settlement intentions caused by air pollution. We found that air pollution has a significant negative effect on migrants’ settlement intention. Air pollution is selective for migrants, and migrants with high income and high education levels are more likely to leave the cities with severe air pollution, while migrants with low education levels and income have less ability to leave their current city of residence, so they are more likely stay in highly polluted areas.

In our findings, the negative effect of air pollution on migrants’ settlement intention is small. This is because China’s urbanization process is not yet fully completed. At China’s current level of urbanization, job opportunity and wage still play an important role in people’s migration decisions and settlement intentions. However, in recent years, the impact of air pollution on migration decisions and settlement intentions has gradually begun to emerge as the public’s increased awareness of the environment and increased concern about air quality. Although the negative effect of air pollution on migrants is not particularly large at this stage, it cannot be ignored that in some cities with high air pollution there has been a loss of population. In particular, migrants with high education levels and high incomes are more likely to leave cities with serious air pollution [36]. As migrants’ income and pursuit of high quality of life, the negative effect of air pollution on migrants’ settlement intention will become greater. The negative effect of air pollution on the population has become a problem that cannot be ignored.

This paper implies that migrants are more and more an important element of economic development and overall national cohesion. Currently, most Chinese cities are now introducing policies aimed at attracting talented internal migrants to settle by offering elevated incomes and a high standard of living. As an important factor of urban livability, air quality is becoming an increasingly significant factor in migrants’ settlement intentions. In particular, more educated and high-income migrants pay greater attention to air quality. They are more likely to consider the hazards it poses to the physical and psychological health of themselves and their families. When facing economic development competition, cities should therefore work toward energy conservation, emission reduction, environmental protection, and air pollution control, and vigorously develop clean energy sources. To avoid population loss and attract highly skilled migrants, local governments should enhance their environmental policies, establish and improve their environmental management systems, and create more livable cities. This will allow the cities to improve their talent agglomeration and boost regional economic development.

This paper has established that air pollution has a significant negative effect on migrants. Still, it is limited because the paper does not ascertain which cities migrants move to when they leave the current city. We intend to explore such issues in subsequent research and further enhance the literature on the effects of air pollution on migration and the settlement intentions of migrants.

## Figures and Tables

**Table 1:** Variable descriptions.

Variable	Description	Mean	Std. Dev.
Settle	Do you intend to live in the area long-term (5 years)? Yes = 1, No = 0	0.790	0.407
AQI	Air quality index of destination city	83.965	21.802
Gdp	Logarithm of GDP per capital	11.345	0.433
Pop	Logarithm of the total population	6.256	0.832
Med	Number of hospital beds per 100 people	6.424	2.028
Uni	Number of students in institutions of higher education per 10,000 people	11.938	1.393
Str	Proportion of tertiary industry in GDP (%)	55.489	11.985
Pri	Ratio of average urban house prices to household income	1.253	0.635
Gen	Genders of migrants: Male = 1, Female = 0	0.477	0.500
Age	Ages of migrants	36.203	10.732
*Hukou*	Rural = 0; Urban = 1	0.209	0.407
Mar	Marital Status: Married = 1; Unmarried = 0	0.828	0.376
Edu	Never went to school = 1; Primary school = 2; Junior high school = 3; Senior high school = 4; College degree = 5; Postgraduate = 6	3.431	0.989
Inc	Logarithm of personal incomes	8.187	0.612
Time	The time lived in the city	5.857	5.657
Ran	Migration ranges	1.724	0.768
Reas	Reasons for Migration: Work = 1; Business = 2; Follow family members = 3; Marriage = 4; Demolition and removal = 5; Seeking refuge = 6; Education and training = 7; Joining the army = 8; Born = 9; Provide for the aged = 10; Other = 11	1.995	3.852

Note: *Hukou* refers to the registered permanent residence system in China.

**Table 2:** The impact of air pollution on migrants’ settlement intention.

	(1)	(2)	(3)	(4)
	Ols	Probit	IV Probit	IV Probit
AQI	0.0002 ***	0.0011 ***	−0.0632 ***	
	(5.74)	(6.35)	(−75.75)	
*L.*AQI				−0.0542 ***
				(−103.87)
Gdp	0.0259 ***	0.1192 ***	0.2554 ***	0.2567 ***
	(10.62)	(12.12)	(27.45)	(29.73)
Med	0.0050 ***	0.0154 ***	0.0067 ***	0.0112 ***
	(8.98)	(7.01)	(3.84)	(6.75)
Uni	0.0139 ***	0.0484 ***	0.1566 ***	0.0956 ***
	(12.91)	(11.08)	(45.68)	(30.01)
Str	0.0006 ***	0.0030 ***	0.0073 ***	0.0070 ***
	(6.95)	(8.46)	(27.68)	(27.77)
Pop	−0.0220 ***	−0.0835 ***	−0.4569 ***	−0.5077 ***
	(−13.74)	(−12.78)	(−49.56)	(−63.23)
Pri	−0.0167 ***	−0.0594 ***	−0.3185 ***	−0.2983 ***
	(−10.76)	(−9.75)	(−61.70)	(−65.53)
Constant	−0.7301 ***	−5.1008 ***	0.1374	0.5628 ***
	(−25.63)	(−44.37)	(0.99)	(3.98)
Individual Control Variable	Yes	Yes	Yes	Yes
Year Fixed Effect	Yes	Yes	Yes	Yes
Observations	243,253	243,253	243,253	242,714
First Stage, Explained Variable: AQI				
Ventilation Coefficients			−0.0019 ***	−0.0019 ***
			(−27.52)	(−22.08)
F-statistics			135,614.06	191,793.05
Wald test			932.10	895.44
Fixed Effect			Yes	Yes

Note: The Z-statistic is in parentheses; *** represent significance levels of 1 percent; at the individual level, the control variables are age, gender, education, reasons for mobility, mobility time, mobility range, household registration status, marital status, and personal income. These rules also apply to subsequent tables.

**Table 3:** The impact of air pollution on heterogeneous migrants.

	(1)		(2)
	Education		Income
AQI	−0.0653 ***	AQI	−0.0195 ***
	(−11.06)		(−8.03)
AQI × edu	−0.0123 ***	AQI × inc	−0.0173 ***
	(−6.99)		(−2.66)
Individual Control Variable	Yes	Individual Control Variable	Yes
City Control Variable	Yes	City Control Variable	Yes
Year Fixed Effect	Yes	Year Fixed Effect	Yes
Observations	243,253	Observations	243,253

Note: The Z-statistic is in parentheses; *** represent significance levels of 1 percent.

**Table 4:** Robustness test.

	**(1)**	**(2)**	**(3)**
	IV Probit	Education	Income
Days	−0.0026 ***	−0.0445 ***	−0.0182 ***
	(−8.11)	(−10.09)	(−12.26)
Days × edu		−0.0116 ***	
		(−10.45)	
Days × inc			−0.0142 ***
			(−12.40)
Individual Control Variable	Yes	Yes	Yes
City Control Variable	Yes	Yes	Yes
Year Fixed Effect	Yes	Yes	Yes
Observations	243,253	243,253	243,253

Note: The Z-statistic is in parentheses; *** represent significance levels of 1 percent.

**Table 5:** The impact of air pollution on heterogeneous migrants.

	**(1)**	**(2)**	**(3)**
	Age	Gender	Marital
AQI	−0.0251 ***	−0.0467 ***	−0.0558 ***
	(−7.12)	(−11.45)	(−22.01)
AQI × age	−0.0011 ***		
	(−4.01)		
AQI × gen		−0.0024 ***	
		(−4.77)	
AQI × mar			−0.0037 ***
			(−6.80)
Individual Control Variable	Yes	Yes	Yes
City Control Variable	Yes	Yes	Yes
Year Fixed Effect	Yes	Yes	Yes
Observations	243,253	243,253	243,253

Note: The Z-statistic is in parentheses; *** represent significance levels of 1 percent.

## Data Availability

The original data of this study was obtained by submitting a formal application to Migrant Population service Center, National Health Commission P.R. China on the website http://www.chinaldrk.org.cn (accessed on 14 April 2022).
